# E2F1-miR-20a-5p/20b-5p auto-regulatory feedback loop involved in myoblast proliferation and differentiation

**DOI:** 10.1038/srep27904

**Published:** 2016-06-10

**Authors:** Wen Luo, Guihuan Li, Zhenhua Yi, Qinghua Nie, Xiquan Zhang

**Affiliations:** 1 Department of Animal Genetics, Breeding and Reproduction, College of Animal Science, South China Agricultural University, Guangzhou 510642, Guangdong Province, China; 2 Guangdong Provincial Key Lab of Agro-Animal Genomics and Molecular Breeding, and Key Lab of Chicken Genetics, Breeding and Reproduction, Ministry of Agriculture, South China Agricultural University, Guangzhou 510642, Guangdong Province, China

## Abstract

miR-17 family microRNAs (miRNAs) are crucial for embryo development, however, their role in muscle development is still unclear. miR-20a-5p and miR-20b-5p belong to the miR-17 family and are transcribed from the *miR-17~92* and *miR-106a~363* clusters respectively. In this study, we found that miR-20a-5p and miR-20b-5p promoted myoblast differentiation and repressed myoblast proliferation by directly binding the 3′ UTR of E2F transcription factor 1 (*E2F1*) mRNA. E2F1 is an important transcriptional factor for organism’s normal development. Overexpression of *E2F1* in myoblasts promoted myoblast proliferation and inhibited myoblast differentiation. Conversely, *E2F1* inhibition induced myoblast differentiation and repressed myoblast proliferation. Moreover, E2F1 can bind directly to promoters of the *miR-17~92* and *miR-106a~363* clusters and activate their transcription, and E2F1 protein expression is correlated with the expression of pri-miR-17~92 and pri-miR-106a~363 during myoblast differentiation. These results suggested an auto-regulatory feedback loop between E2F1 and miR-20a-5p/20b-5p, and indicated that miR-20a-5p, miR-20b-5p and E2F1 are involved in myoblast proliferation and differentiation through the auto-regulation between E2F1 and miR-20a-5p/20b-5p. These findings provide new insight into the mechanism of muscle differentiation, and further shed light on the understanding of muscle development and muscle diseases.

MicroRNAs (miRNAs) are endogenous noncoding single-stranded RNAs that can play important regulatory roles in animals and plants^1^. Approximately one-third of all mammalian genes are thought to be targeted by miRNAs^2^. Many physiological and biochemical processes, such as tumorigenesis, development, cell proliferation, differentiation and apoptosis, have been found to be regulated by miRNAs^3^,^4^,^5^,^6^,^7^. During muscle development, many miRNAs have been found to regulate diverse aspects of developmental processes. Some of these miRNAs exhibit muscle-specific expression pattern and play a critical role in muscle cell proliferation and differentiation. These muscle-specific miRNAs are called myogenic miRNAs (MyomiRs), which include miR-1, miR-206 and miR-133^8^,^9^. They can interact with myogenic regulatory factors (MRFs) and regulate many muscle development-related genes, therefore play important roles in muscle development^4^,^10^. Additionally, many non-MyomiRs also play roles in muscle development. By directly inhibiting *MyoD, MEF2C, Pax3, YY1, IGF-2, Cdc25A* and the other genes that are related to myoblast proliferation or differentiation, these non-MyomiRs are all involved in the regulation of muscle development^4^,^11^,^12^,^13^,^14^,^15^.

Three paralogous miRNA gene clusters, which include *miR-17~92, miR-106a~363* and *miR-106b~25*, are implicated in a wide variety of cellular processes^16^. Mice deficient for *miR-17~92* die shortly after birth with defects in lung, heart and other organs, while ablation of *miR-106a~363* or *miR-106b~25* has no obvious phenotypic consequences^17^. Among these parologous miRNAs, the miR-17 family, composed of miR-17, miR-20a/b, miR-106a/b and miR-93, has been demonstrated to involve in critical pathways that regulate embryo development and stem cell differentiation^18^. Members of the miR-17 family have same seed sequence, therefore these paralogous miRNAs would sharing some overlapping functions. In human tumours, the expression of miR-17 family miRNAs is correlated with the cell cycle genes expression, and this correlation is resulted from the inhibitory effect of these miRNAs to their common target genes, which can negatively regulate cell cycle progression^19^. miR-17-5p and miR-20a, which are induced by c-Myc, can regulate E2F1 translational yield by binding to the 3′ UTR of *E2F1* mRNA and then regulate cell proliferation^20^. Additionally, the developmental processes of lung, heart and B cell are all related to the roles of the miR-17 family miRNAs^16^, demonstrating the critical combined effects of these miRNAs during embryo development. However, as an essential part of embryo development regulation, the roles of miR-17 family during muscle development are still not clear.

Members of the E2F transcription factor family play an essential role in the regulation of cellular proliferation and cell cycle progression during organism’s normal development^21^,^22^. Mice lacking E2F1 show testicular atrophy and exocrine gland dysplasia^23^. During muscle development, dyregulated E2F1 expression can induce the inhibition of myogenic differentiation^24^. E2F1-mediated transcription plays an essential role in myogenesis^25^. The transcription activity of myogenic bHLH proteins MyoD and myogenin, which are critical regulators for myogenic differentiation^26^,^27^, can be inhibited by E2F1^25^. During C2C12 myocyte differentiation, the expression of *E2F1* is down-regulated^24^. However, the regulation of *E2F1* during this process still remains to be demonstrated.

Avian is an ideal model organism for muscle development research. In our previous study, we found that miR-20a-5p and miR-20b-5p have a significantly higher expression level in skeletal muscle at day 14 embryo (E14) than at 7 week (7W) chick^28^. Considering that the proliferation and differentiation of muscle cells are very different between E14 and 7W, we assume that miR-20a-5p and miR-20b-5p may be involved in these two processes. In this study, we confirmed that miR-20a-5p and miR-20b-5p can regulate myoblast proliferation and differentiation in quail muscle clone 7 (QM-7) cells, which closely resemble their mammalian counterparts in most respects^29^,^30^. miR-20a-5p and miR-20b-5p overexpression inhibited myoblast proliferation but promoted myoblast differentiation, whereas inhibition of miR-20a-5p and miR-20b-5p could result in increased proliferation and decreased differentiation of myoblasts. Both miR-20a-5p and miR-20b-5p can directly bind to the 3′ UTR of *E2F1* mRNA and inhibit E2F1 expression. Moreover, E2F1 binds directly to the promoters of the *miR-17~92* and *miR-106a~363* clusters and activates their transcription. The protein level of E2F1 is correlated with the expression of pri-miR-17~92 and pri-miR-106a~363 during myoblast differentiation. Altogether, these results suggest an auto-regulatory feedback loop between E2F1 and miR-20a-5p/20b-5p, and this auto-regulatory loop may play important roles in myoblast proliferation and differentiation.

## Results

### miR-20a-5p and miR-20b-5p repress myoblast proliferation

**Figure 1:**
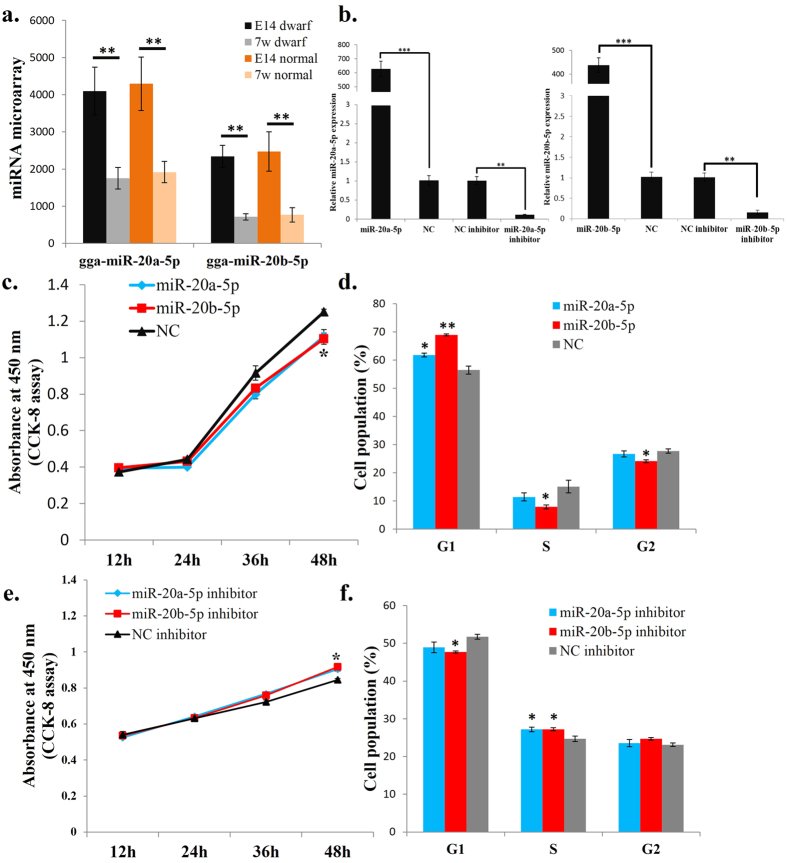
miR-20a-5p and miR-20b-5p repress myoblast proliferation. (**a**) The miRNA microarray hybridization signals of miR-20a-5p and miR-20b-5p in E14 and 7w of dwarf and normal chickens’ leg muscle. (**b**) The expression of miR-20a-5p and miR-20b-5p after transfection of the indicated miRNA mimic or miRNA inhibitor. (**c**) CCK-8 assay was performed to access the effect of miR-20a-5p and miR-20b-5p overexpression on myoblast proliferation. (**d**) Cell cycle analysis of myoblasts at 48 h after transfection of miR-20a-5p and miR-20b-5p mimics or NC mimic. (**e**) CCK-8 assay was performed to access the effect of miR-20a-5p and miR-20b-5p loss-of-function on myoblast proliferation. (**f**) Cell cycle analysis of myoblasts at 48 h after transfection of miR-20a-5p inhibitor, miR-20b-5p inhibitor or NC inhibitor. Results are shown as the mean ± sem of three independent experiments. One sample *t* test was used to analysis the statistical differences between groups. *p < 0.05; **p < 0.01; ***p < 0.001.

Our previous microarray data (GSE37360, GSE37367 and GSE37368) showed that the expression levels of miR-20a-5p and miR-20b-5p are significantly higher in the leg muscle of E14 normal and dwarf chickens than those in 7w chickens (Fig. 1a). To investigate the effects of miR-20a-5p and miR-20b-5p on avian muscle cell, we used the QM-7 cell lines as a model system to identify the functional characteristics of miR-20a-5p and miR-20b-5p in skeletal myogenesis. Firstly we introduced miR-20a-5p (5′-UAAAGUGCUUAUAGUGCAGGUAG-3′) and miR-20b-5p (5′-CAAAGUGCUCAUAGUGCAGGUAG-3′) mimics into the QM-7 cells (Fig. 1b) and then monitored the proliferation status of cells using the Cell Counting Kit-8. The results showed that overexpression of either miR-20a-5p or miR-20b-5p repressed cell proliferation significantly (Fig. 1c). Next, we used flow cytometry analysis of the cell cycle to test the effects of miR-20a-5p and miR-20b-5p on cell cycle regulation. The results showed that QM-7 transfected with the miR-20a-5p and miR-20b-5p mimic could significantly increase the number of cells in the G0/G1 stage, but decrease the number of cells in the S and G2 stages (Fig. 1d and Supplementary File 1). Additionally, we transfected QM-7 cells with the inhibitor of miR-20a-5p and miR-20b-5p (Fig. 1b). CCK-8 assay showed that either miR-20a-5p or miR-20b-5p knockdown can promote cell proliferation (Fig. 1e), and the results of cell cycle analysis showed that both miR-20a-5p inhibitor and miR-20b-5p inhibitor can increase the number of cells in the S stage (Fig. 1f and Supplementary File 1). Therefore, these data suggest that both miR-20a-5p and miR-20b-5p can repress myoblast proliferation and affect cell cycle progression of avian myoblasts.

### miR-20a-5p and miR-20b-5p promote myoblast differentiation

**Figure 2:**
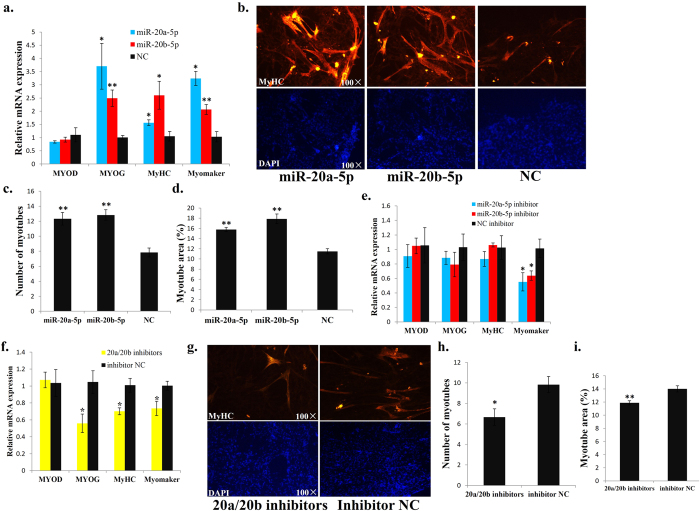
miR-20a-5p and miR-20b-5p promote myoblast differentiation. (**a**) The relative mRNA expression of *MYOD, MYOG, MyHC* and *Myomaker* after transfection of miR-20a-5p, miR-20b-5p or NC mimic. (**b**) MyHC staining of myoblasts at 72 h after transfection of miR-20a-5p, miR-20b-5p or NC mimic. (**c**) Number of myotubes at 72 h after transfection of miR-20a-5p, miR-20b-5p or NC mimic. (**d**) Myotube area (%) at 72 h after transfection of miR-20a-5p, miR-20b-5p or NC mimic. (**e**) The relative mRNA expression of *MYOD, MYOG, MyHC* and *Myomaker* after transfection of miR-20a-5p inhibitor, miR-20b-5p inhibitor or NC inhibitor. (**f**) The relative mRNA expression of *MYOD, MYOG, MyHC* and *Myomaker* after transfection of miR-20a-5p/miR-20b-5p mixed inhibitors or NC inhibitor. (**g**) MyHC staining of myoblasts at 72 h after transfection of miR-20a-5p/miR-20b-5p mixed inhibitors or NC inhibitor. (**h**) Number of myotubes at 72 h after transfection of miR-20a-5p/miR-20b-5p mixed inhibitors or NC inhibitor. (**i**) Myotube area (%) at 72 h after transfection of miR-20a-5p/miR-20b-5p mixed inhibitor or NC inhibitor. Results are shown as the mean ± sem of three independent experiments. One sample *t* test was used to analysis the statistical differences between groups. *p < 0.05; **p < 0.01.

As cell cycle arrest is important for myoblast differentiation, and both miR-20a-5p and miR-20b-5p are implicated in cell cycle arrest, we next studied the potential functions of miR-20a-5p and miR-20b-5p in myoblast differentiation. QM-7 cells cultured in growth medium (GM) were transfected with the miR-20a-5p mimic and miR-20b-5p mimic. After transfection, the cells were induced to differentiation by changing the GM to differentiation medium (DM). Four major myoblast differentiation marker genes, *MYOD* (named *mf1* gene in quail, accession: L16686.1), *MYOG* (named *mf2* gene in quail, accession: L15473.1), *MyHC* (named *slow myosin heavy chain* gene in quail, accession: U53862.1) and *Myomaker* (referred to chicken *TMEM8C* gene, accession: KP230536.1), were detected at 72 h after differentiation according to qPCR analysis. The expressions of *MYOG, MyHC* and *Myomaker* were all significantly up-regulated in the miR-20a-5p- and miR-20b-5p-transfected cells compared with those in the control cells (Fig. 2a), whereas the *MYOD* expression did not alter between the two groups of cells (Fig. 2a). Moreover, overexpression of miR-20a-5p and miR-20b-5p markedly increased the number of MyHC-positive cells (Fig. 2b,c) and the total areas of myobube (Fig. 2d).

To better understand the physiological relevance of miR-20a-5p and miR-20b-5p during myoblast differentiation, we transfected the QM-7 cells with miR-20a-5p inhibitor and miR-20b-5p inhibitor and then induced the cells to differentiation. After 72 h differentiation, we detected the relative expression of the four genes. The results showed that only *Myomaker* was significantly down-regulated in the miR-20a-5p inhibitor- and miR-20b-5p inhibitor-transfected cells (Fig. 2e). However, as we co-transfected the QM-7 cells with miR-20a-5p and miR-20b-5p mixed inhibitors and then induced the cells to differentiation, the expressions of *MYOG, MyHC* and *Myomaker* were all reduced when compared with those in the control cells, although the *MYOD* expression also has no differences between the two groups of cells (Fig. 2f). The number of MyHC-positive cells and the total areas of myotubes were also significantly reduced when miR-20a-5p and miR-20b-5p mix inhibitors were transfected during QM-7 differentiation (Fig. 2g–i). Therefore, we argue that both miR-20a-5p and miR-20b-5p promote myoblast differentiation.

### *E2F1* is a direct target gene of miR-20a-5p and miR-20b-5p

**Figure 3:**
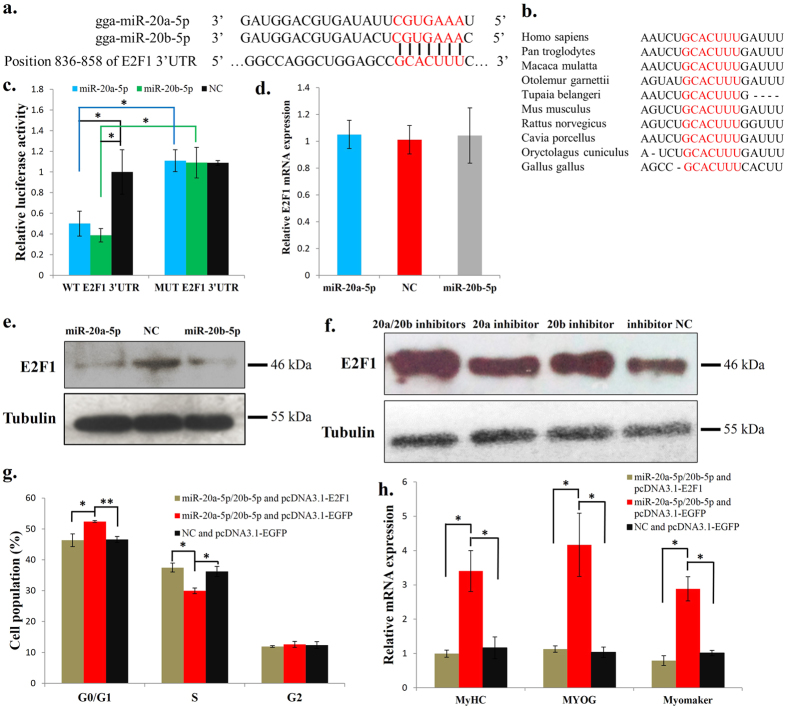
*E2F1* is a direct target gene of miR-20a-5p and miR-20b-5p. (**a**) The potential binding site (red) of miR-20a-5p and miR-20b-5p in the *E2F1* mRNA 3′ UTR. Sequence in blue indicate the mutation of the binding site. (**b**) The potential binding site (red) of miR-20a-5p and miR-20b-5p in the *E2F1* mRNA 3′ UTR is highly conserved among vertebrates. (**c**) Dual-luciferase reporter assay indicated that miR-20a-5p and miR-20b-5p can bind to the predicted binding site in the *E2F1* mRNA 3′ UTR. (**d**) *E2F1* mRNA expression after transfection of miR-20a-5p and miR-20b-5p mimic. (**e**) E2F1 protein expression after transfection of miR-20a-5p and miR-20b-5p mimic. (**f**) E2F1 protein expression after transfection of miR-20a-5p inhibitor, miR-20b-5p inhibitor, miR-20a-5p/miR-20b-5p mixed inhibitors or NC inhibitor. (**g**) Myoblasts were co-transfected with miR-20a-5p/20b-5p and pcDNA3.1-E2F1, miR-20a-5p/20b-5p and pcDNA3.1-EGFP, or NC and pcDNA3.1-EGFP as control, followed by 48 h culture, and the cell cycle phase was then analysed. (**h**) Myoblasts were co-transfected with miR-20a-5p/20b-5p and pcDNA3.1-E2F1, miR-20a-5p/20b-5p and pcDNA3.1-EGFP, or NC and pcDNA3.1-EGFP as control, followed by 72 h differentiation, and the expression of *MYOG, MyHC* and *Myomaker* was then quantified by q-PCR. Results are shown as the mean ± sem of three independent experiments. One sample *t* test was used to analysis the statistical differences between groups. *p < 0.05; **p < 0.01.

Previous study has demonstrated that *E2F1* is a direct target of miR-20a in a mammal prostate cancer cell line^31^, and the E2F1 transcription factor is an important regulator of embryo development^23^. Therefore, we examined whether *E2F1* is a target gene of miR-20a-5p and miR-20b-5p during avian myoblast differentiation. By searching for the 3′ UTR of chicken *E2F1* mRNA (NM_205219.1), we found that the seed sequence of miR-20a-5p and miR-20b-5p can perfectly match the position 851-857 of chicken *E2F1* mRNA 3′ UTR (Fig. 3a), and the sequence of this position are highly conserved among vertebrates (Fig. 3b). To further determine whether miR-20a-5p and miR-20b-5p could bind to the 3′ UTR of *E2F1* mRNA directly, we constructed two dual-luciferase reporter vectors with the wild-type or mutant 3′ UTR of *E2F1* inserted at the 3′ end of the firefly luciferase gene. Then we tested the effects of miR-20a-5p and miR-20b-5p on these reporters in DF-1 cells. The results showed that miR-20a-5p and miR-20b-5p transfection significantly inhibited the reporter activity, whereas they had no effect on the mutant reporter activity (Fig. 3c), suggesting that the predicted binding site in the *E2F1* 3′ UTR is a bona fide target of miR-20a-5p and miR-20b-5p. The mRNA and protein levels of *E2F1* were also detected in miR-20a-5p and miR-20b-5p overexpressing myoblasts, respectively. Myoblasts transfected with either miR-20a-5p or miR-20b-5p would reduce E2F1 protein expression, whereas they had no significant effect on the mRNA level of *E2F1* (Fig. 3d,e). Additionally, E2F1 protein level can be increased with the transfection of either miR-20a-5p inhibitor or miR-20b-5p inhibitor (Fig. 3f), and the protein level can be increased more when miR-20a-5p and miR-20b-5p inhibitors were co-transfected into the cells (Fig. 3f).

To further evaluate the principal roles of E2F1 in the functional effects induced by miR-20a-5p and miR-20b-5p, we overexpressed E2F1 in myoblasts with transfection of miR-20a-5p and miR-20b-5p mixed mimics. The results showed that E2F1 overexpression can restore cell cycle arrest in miR-20a-5p/20b-5p transfected cells (Fig. 3g), and the presence of miR-20a-5p/20b-5p with control plasmid (pcDNA3.1-EGFP) in myoblasts led to increased cell cycle arrest in the G0/G1 stage. Furthermore, E2F1 overexpression can also overcome the differentiation effects of miR-20a-5p/20b-5p in myoblasts. The increased expressions of *MYOG, MyHC* and *Myomaker* as a consequence of miR-20a-5/20b-5p transfection were attenuated by the co-expression of E2F1 (Fig. 3h). Therefore, *E2F1* is a direct target gene of miR-20a-5p and miR-20b-5p, and its overexpression could overcome the cell cycle and differentiation effects of miR-20a-5p and miR-20b-5p in myoblasts.

### E2F1 promotes myoblast proliferation and represses myogenic differentiation

**Figure 4:**
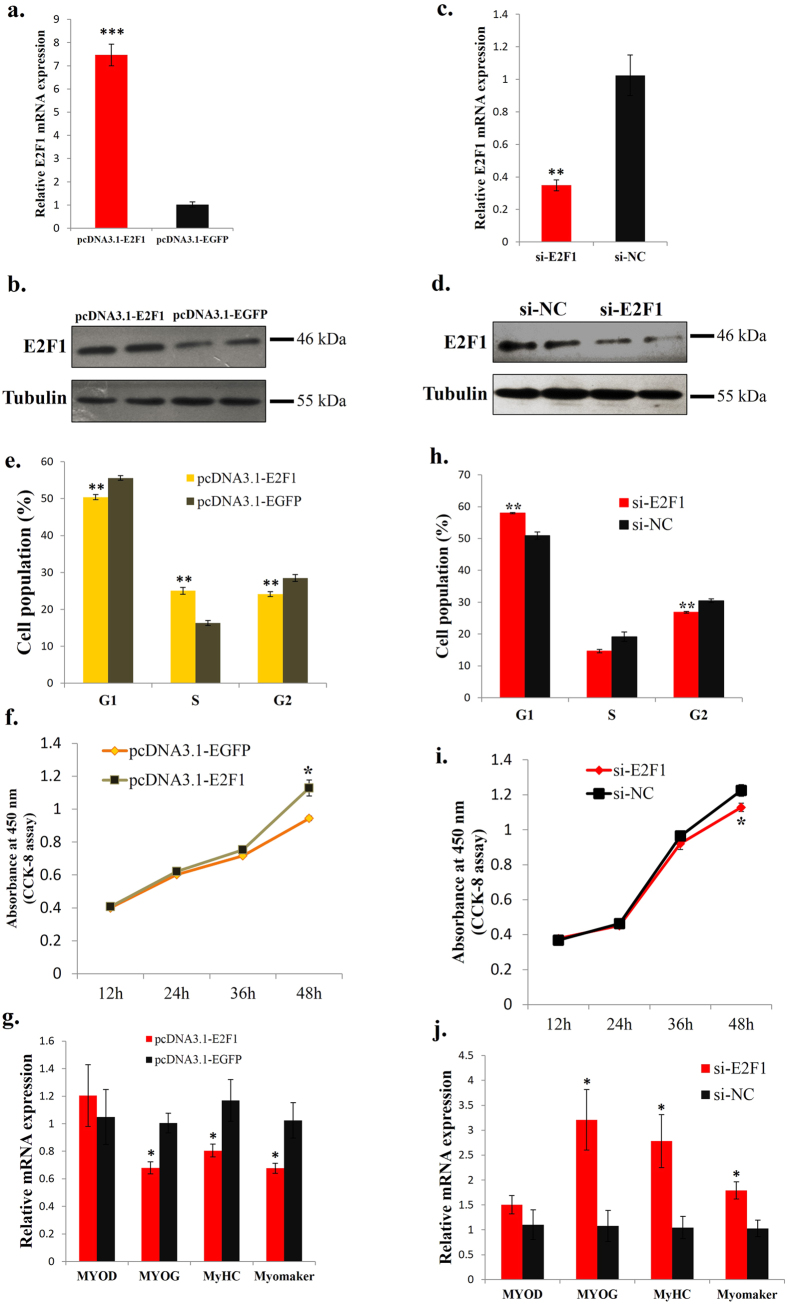
E2F1 promotes myoblast proliferation and represses myogenic differentiation. (**a**) *E2F1* mRNA expression after pcDNA3.1-E2F1 and pcDNA3.1-EGFP transfection. (**b**) E2F1 protein expression after pcDNA3.1-E2F1 and pcDNA3.1-EGFP transfection. (**c**) *E2F1* mRNA expression after si-E2F1 and si-NC transfection. (**d**) E2F1 protein expression after si-E2F1 and si-NC transfection. (**e**) Cell cycle analysis of myoblasts at 48 h after pcDNA3.1-E2F1 and pcDNA3.1-EGFP transfection. (**f**) CCK-8 assay was performed to access the effects of E2F1 overexpression on myoblast proliferation. (**g**) The relative mRNA expression of *MYOD, MYOG, MyHC* and *Myomaker* after pcDNA3.1-E2F1 and pcDNA3.1-EGFP transfection. (**h**) Cell cycle analysis of myoblasts at 48 h after si-E2F1 and si-NC transfection. (**i**) CCK-8 assay was performed to access the effects of E2F1 loss-of-function on myoblast proliferation. (**j**) The relative mRNA expression of *MYOD, MYOG, MyHC* and *Myomaker* after si-E2F1 and si-NC transfection. Results are shown as the mean ± sem of three independent experiments. One sample *t* test was used to analysis the statistical differences between groups. *p < 0.05; **p < 0.01; ***p < 0.001.

In this study, we performed *E2F1* overexpression and knockdown assays in QM-7 cells to verify its functions in avian myogenic proliferation and differentiation. As shown in Fig. 4, transfecting pcDNA3.1-E2F1 into myoblasts increased *E2F1* mRNA and protein expression (Fig. 4a,b), whereas transfecting si-E2F1 into myoblasts decreased *E2F1* mRNA and protein expression (Fig. 4c,d). *E2F1* overexpression significantly promoted myoblast proliferation, reduced the number of cells that progressed to G0/G1 phase and increased the number of cells that progressed to S phase (Fig. 4e,f and Supplementary File 1). Additionally, *E2F1* overexpression significantly reduced *MYOG, MyHC* and *Myomaker* expression (Fig. 4g), suggesting an inhibitory role of E2F1 in myogenic differentiation. On the other hand, knockdown of the *E2F1* expression in QM-7 cells significantly increased the number of cells in the G0/G1 stage and reduced the number of cells in the S and G2 stages (Fig. 4h and Supplementary File 1). The CCK-8 assay also showed that *E2F1* knockdown significantly reduced cell proliferation (Fig. 4i). Moreover, we transfected QM-7 cells with anti-E2F1 siRNA in GM and then induced the cell to differentiation. The results showed that *E2F1* knockdown significantly increased *MYOG, MyHC* and *Myomaker* expression, whereas the expression of *MYOD* had not been affected (Fig. 4j). Therefore we conclude that E2F1 can regulate avian myoblast proliferation and differentiation.

### E2F1 regulates *miR-17~92* and *miR-106a~363* expression directly

**Figure 5:**
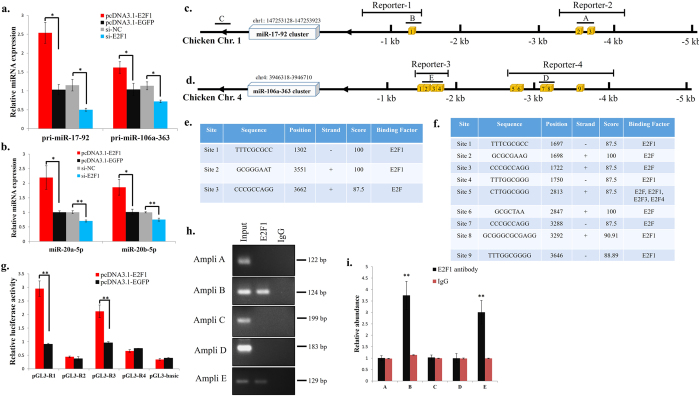
E2F1 regulates *miR-17~92* and *miR-106a~363* expression directly. (**a**) The relative expression of pri-miR-17~92 and pri-miR-106a~363 after transfection with indicated plasmid or siRNA. (**b**) The relative expression of miR-20a-5p and miR-20b-5p after transfection with the indicated plasmid or siRNA. (**c**) Location of the putative E2F binding sites in the *miR-17~92* cluster promoter. Yellow boxes represent the putative E2F binding sites. Horizontal bars represent the amplicons A to C used in the ChIP assay. Reporter-1 and Reporter-2 represent the promoter regions used in luciferase reporter assay. (**d**) Location of the putative E2F binding sites in the *miR-106a~363* cluster promoter. Yellow boxes represent the putative E2F binding sites. Horizontal bars represent the amplicons D and E used in the ChIP assay. Reporter-3 and Reporter-4 represent the promoter regions used in luciferase reporter assay. (**e**) Basic information of the predicted E2F binding sites in the *miR-17~92* cluster promoter. (**f**) Basic information of the predicted E2F binding sites in the *miR-106a~363* cluster promoter. (**g**) The relative luciferase activity of pGL3-R1, pGL3-R2, pGL3-R3, pGL3-R4 and pGL3-Basic after pcDNA3.1-E2F1 or pcDNA3.1-EGFP transfection. (**h**) ChIP and PCR amplification of regions from the *miR-17~92* and *miR-106a~363* cluster promoter. (**i**) Quantitation of the ChIP assay results by real-time PCR. Results are shown as the mean ± sem of three independent experiments. One sample *t* test was used to analysis the statistical differences between groups. *p < 0.05; **p < 0.01.

When we transfected QM-7 cells with pcDNA3.1-E2F1 or anti-E2F1 siRNA, we found that the expression of both *miR-17~92* and *miR-106a~363* pri-precursors was significantly changed (Fig. 5a). Additionally, the mature miR-20a-5p and miR-20b-5p expression also significantly changed (Fig. 5b). Considering that E2F1 is a transcriptional factor, we explored the possibility that E2F1 may directly regulate the transcription of the *miR-17~92* and *miR-106a~363* clusters. By using MATCH online software, a tool for predicting transcription factor binding sites in DNA sequence^32^, we found that there are three and nine putative E2F-binding sites within the first 5 kb of the promoters of the chicken *miR-17~92* cluster and *miR-106a-363* cluster, respectively (Fig. 5c–f). To confirm that E2F1 can regulate the expression of *miR-17~92* and *miR-106a~363* clusters directly, we cloned four reporters containing the potential E2F-binding sites of these promoters into the pGL3-basic luciferase reporter plasmid. The luciferase activity of both reporter-1 and reporter-3 was significantly increased when the two reporters were co-transfected with pcDNA3.1-E2F1 plasmid, respectively (Fig. 5g). However, reporter-2 and reporter-4 did not alter their luciferase activities when the pcDNA3.1-E2F1 plasmid were co-transfected, respectively (Fig. 5g). These results suggested that E2F1 can directly bind the regions of reporter-1 and reporter-3 and activate their transcriptions.

To further confirm the binding of endogenous E2F1 to the promoters, we performed chromatin immunoprecipitation (ChIP) experiments to detect the association of E2F1 with the *miR-17~92* and *miR-106a-363* promoters in chicken primary myoblasts. In the *miR-17~92* promoter, we generated amplicon A which overlaps site 2 and 3, amplicon B which overlaps site 1, and amplicon C which was used as a negative control. In the miR-106a~363 promoter, we generated amplicon D which overlaps site 7 and 8, and amplicon E which overlaps site 1, 2, 3 and 4 (Fig. 5c,d). The ChIP-PCR showed that only amplicon B and amplicon E were amplified after ChIP assay with anti-E2F1 antibody (Fig. 5h), and the relative abundance of the PCR product was more than two-fold compared to the control group (Fig. 5i). On the other hand, amplicon A, amplicon C and amplicon D were all negative. Therefore, these results suggest that the fragments of the amplicon B and amplicon E contain E2F1 binding site. Altogether, the above results present an auto-regulatory feedback loop, where translation of the *E2F1* mRNA is controlled by miR-20a-5p and miR-20b-5p, which pri-precursors are regulated by E2F1 at the transcriptional level.

### Correlation between pri-miR-17~92, pri-miR-106a~363 and E2F1 protein levels in QM-7 and chicken primary myoblasts undergoing differentiation *in vitro*

**Figure 6:**
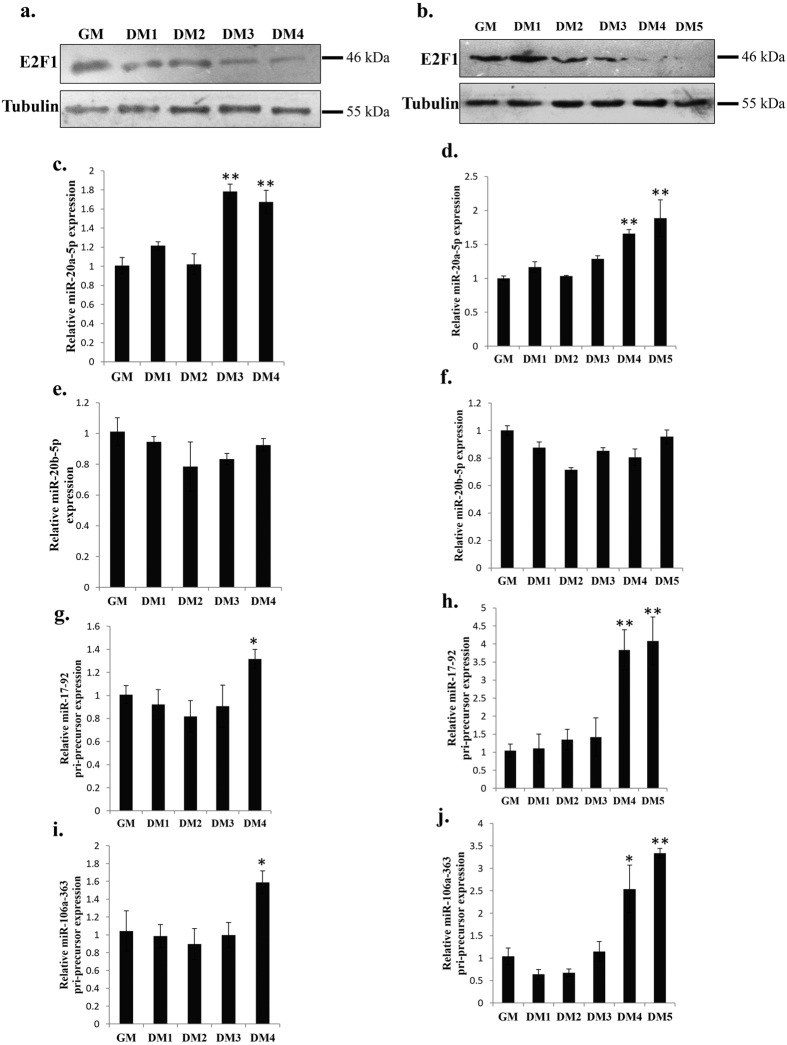
Correlation between pri-miR-17~92, pri-miR-106a~363 and E2F1 protein levels in QM-7 and chicken primary myoblast undergoing differentiation *in vitro*. (**a**) E2F1 protein expression during QM-7 myoblast differentiation. (**b**) E2F1 protein expression during chicken primary myoblast differentiation. (**c**) The relative miR-20a-5p expression during QM-7 myoblast differentiation. (**d**) The relative miR-20a-5p expression during chicken primary myoblast differentiation. (**e**) The relative miR-20b-5p expression during QM-7 myoblast differentiation. (**f**) The relative miR-20b-5p expression during chicken primary myoblast differentiation. (**g**) The relative pri-miR-17~92 expression during QM-7 myoblast differentiation. (**h**) The relative pri-miR-17~92 expression during chicken primary myoblast differentiation. (**i**) The relative pri-miR-106a~363 expression during QM-7 myoblast differentiation. (**j**) The relative pri-miR-106a~363 expression during chicken primary myoblast differentiation. Results are shown as the mean ± sem of three independent experiments. One sample *t* test was used to analysis the statistical differences between groups. *p < 0.05; **p < 0.01.

To better understand the potential physiologic relevance of miR-20a-5p, miR-20b-5p and *E2F1* interaction during myogenesis, we next examined the expression of miR-20a-5p, miR-20b-5p and *E2F1* during QM-7 and chicken primary myoblasts undergoing differentiation *in vitro*. Western blot assays showed that the E2F1 protein level was down-regulated gradually from GM to DM during both QM-7 and primary myoblast differentiation (Fig. 6a,b). On the other hand, miR-20a-5p expression was significantly up-regulated during myoblast differentiation (Fig. 6c,d), and there is no significant change in miR-20b-5p expression during the differentiation process (Fig. 6e,f). For the expression of miRNA pri-precursors, both pri-miR-17~92 and pri-miR-106a~363 were significantly up-regulated during myoblast differentiation (Fig. 6g–j). Therefore, the results of up-regulated pri-miR-17~92 and pri-miR-106a~363 expression and down-regulated E2F1 expression suggest a correlation relationship between these regulators during myoblast differentiation.

## Discussion

**Figure 7:**
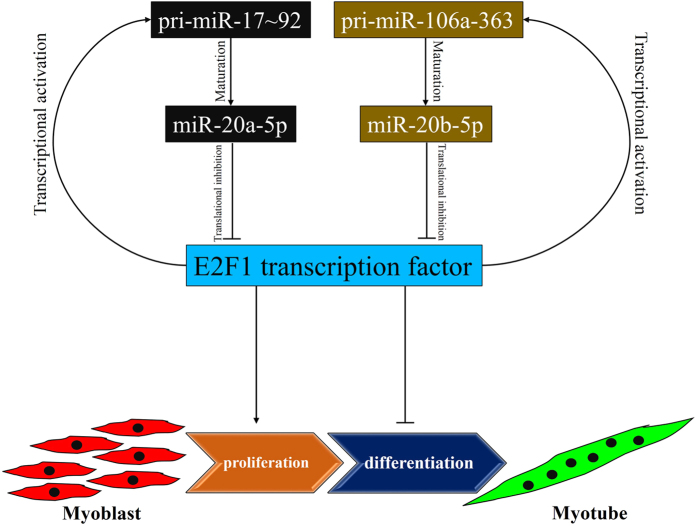
Model for the proposed E2F1-miR-20a-5p/20b-5p auto-regulatory feedback loop in the regulation of myoblast proliferation and differentiation.

In this study, we found that the *miR-17~92* cluster and *miR-106a~363* cluster are directly regulated by the transcription factor E2F1. As miR-20a-5p and miR-20b-5p, which belong to the two clusters respectively, can inhibit E2F1 protein expression, an auto-regulatory feedback loop can be presented between E2F1 and the two clusters (Fig. 7). The auto-regulatory feedback loop is an interesting model for the regulation of gene expression, especially with the participation of miRNAs in this loop. miRNA regulation in this loop not only act as a simple linear regulator, but also contain check-and-balance function^33^. An example of this auto-regulatory loop is miR-15a and its target gene *c-myb*. miR-15a directly binds and regulates *c-myb* expression, while c-Myb protein binding site is occupied in *miR-15a* promoter and is required for miR-15a expression^34^. The presence of miR-15a in this auto-regulatory loop is to regulate *c-myb* expression and to play an importance role in human hematopoiesis^34^. Another example is a muscle-specific miRNA miR-1 and its target gene *Yin Yang 1 (YY1*), which is a repressor of muscle genes^13^. This auto-regulatory feedback loop not only maintain the balance of the expression level of transcription factors and miRNAs, but also is involved in the regulation of myogenesis^13^. In this study, we found another auto-regulatory feedback loop, E2F1-miR-20a-5p/20b-5p, which might also be involved in myogenesis (Fig. 7). Previous study has demonstrated that E2F1 down-regulation is required for myoblast differentiation^24^. Our results have indicated that miR-20a-5p and miR-20b-5p can directly inhibit *E2F1* expression in myoblasts, and miR-20a-5p is up-regulated during myoblast differentiation. Therefore, the auto-regulatory feedback loop of E2F1-miR-20a-5p/20b-5p might play roles in the regulation of E2F1 during myoblast differentiation, specifically in repressing E2F1 expression to relieve the inhibitory role of E2F1 in this process.

Skeletal muscle differentiation is a complex process with the regulation of diverse genetic and environmental factors. These factors can interact with each other and construct multiple signaling pathways to regulate the cell differentiation process. The network between transcription factors and miRNAs is an interesting global feature and has been extensive focused on^35^,^36^. An auto-regulatory feedback loop with transcription factor and miRNA can make the regulatory network more abundant and of highly organization. However, few of these kinds of feedback loops have been found in myogenesis. Additionally, as an important regulator of embryo development, miR-17 family has been rarely studied in myogenesis. Our results not only showed that both miR-20a-5p and miR-20b-5p can regulate the proliferation and differentiation of myoblasts, but also proposed an E2F1-miR-20a-5p/20b-5p auto-regulatory feedback loop that might be involved in myogenesis. These findings provide new information into the regulation of skeletal muscle differentiation.

The E2F-miR-20a auto-regulatory feedback loop not only exists in avian myoblast cell, but also exists in human cell. In a prostate cancer cell line, miR-20a directly inhibits the translation of the *E2F1, E2F2* and *E2F3* mRNA, and the endogenous E2F1, E2F2 and E2F3 bind to the promoter of the *miR-17~92* cluster and activate its transcription^31^. In HEK-293 cells, *miR-17~92* cluster and E2Fs can also construct an auto-regulatory feedback loop^37^. This auto-regulatory feedback loop is important for balancing endogenous E2Fs expression level and may be involved in the regulation of cellular proliferation, transformation and apoptosis^31^,^37^. In this study, we not only found the auto-regulatory feedback loop of E2F1-miR-20a-5p, but also found the auto-regulatory feedback loop of E2F1-miR-20b-5p. Both these feedback loops might be involved in avian myoblast proliferation and differentiation. miR-20a-5p and miR-20b-5p inhibit the protein level of E2F1 directly, and the endogenous E2F1 binds to the promoters of the *miR-17~92* cluster and *miR-106a~363* cluster and activates their transcription. However, there are still some questions existing in myoblast differentiation process. Endogenous E2F1 can bind and activate pri-miR-17~92 and pri-miR-106a~363 expression. The protein level of E2F1 is down-regulated during myoblast differentiation, whereas the expression of pri-miR-17~92 and pri-miR-106a~363 are up-regulated, suggesting that there be another transcriptional factor that can promote pri-miR-17~92 and pri-miR-106a~363 transcription during the differentiation process. Previous studies have demonstrated that c-Myc directly activate transcription of the *miR-17~92* and *miR-106a~363* clusters^20^,^38^. In addition, p53, STAT5 and MYCN can also regulate the transcription of the *miR-17~92* or *miR-106a~363* clusters directly^39^,^40^,^41^,^42^. Therefore, E2F1 is not the only factor that can regulate the transcription of these two clusters. Other transcriptional factors may also be involved in the regulation of *miR-17~92* and *miR-106a~363* clusters transcription during myoblast differentiation.

The protein level of E2F1 is down-regulated during avian myoblast differentiation. Similarly, *E2F1* mRNA level in C2 myocytes is also reduced during differentiation process^24^. E2F1 is important for cell proliferation and development, but the regulation of this protein during muscle differentiation still remains unclear. Our data showed that miR-20a-5p and miR-20b-5p directly regulated E2F1 protein level by binding to the 3′ UTR of *E2F1* mRNA. However, miR-20b-5p expression level was relatively consistent during myoblast differentiation, and the expression of miR-20a-5p was modestly up-regulated. These expression patterns may not fully explain the down-regulated expression of E2F1 protein level. There may be other factors involved in the inhibition of E2F1 protein expression. The members of miR-17 family have same seed sequences, suggesting that these miRNAs might sharing common target genes. Previous studies have demonstrated that miR-17-5p, miR-106a-5p, miR-106b-5p and miR-93-5p can also inhibit E2F1 expression directly^43^,^44^,^45^. Therefore, it is possible that all members of the miR-17 family can directly inhibit E2F1 expression during myoblast differentiation. Considering that pri-miR-17~92 and pri-miR-106a~363 expression are all significantly up-regulated during myoblast differentiation, the down-regulation of E2F1 protein level might be resulted from the up-regulated expression of all of the miR-17 family members.

The inhibitory effect of E2F1 in myogenic differentiation is through the induction of genes which repress the transcription activity of MYOG and MYOD^25^. E2F1 overexpression cannot inhibit *MYOD* expression, but the expression of *MYOG* and *MyHC*, which can be transcribed by MYOD^46^,^47^, would be reduced in E2F1 overexpressed myoblasts^24^. This regulatory effect also exists in our results, which were performed in avian myoblasts. Additionally, either miR-20a-5p or miR-20b-5p is able to regulate *MYOG* and *MyHC* expressions by inhibiting *E2F1* expression directly. However, *MYOG* and *MyHC* expressions were downregulated only when both miR-20a-5p and miR-20b-5p were inhibited. This result might be due to the insufficient release of *E2F1* expression from miR-20a-5p and miR-20b-5p inhibition by only one of the inhibitors transfection. The E2F1 protein level would be reduced more when the inhibitors of miR-20a-5p and miR-20b-5p were co-transfected in myoblasts. Additionally, the potential complementary effect between miR-20a-5p and miR-20b-5p can also influence the effect of only one of the inhibitors transfection. On the other hand, Myomaker is an important transmembrane protein in myoblast fusion^48^,^49^,^50^. Its expression during myoblast differentiation depends on the transcription activity of MYOD and MYOG^49^,^51^. In this study, inhibition of miR-20a-5p and miR-20b-5p cannot reduce myogenic differentiation, but the myoblast fusion might be reduced based on the down-regulation of *Myomaker*. As E2F1 expression is reduced in the transfection of one of the miRNA inhibitors, the transcription activity of both *MYOD* and *MYOG* would be inhibited. Therefore, *Myomaker* expression can also be down-regulated in the case that only one of the inhibitors was transfected.

In conclusion, both miR-20a-5p and miR-20b-5p inhibit myoblast proliferation and promote myoblast differentiation by directly binding and inhibiting *E2F1* mRNA expression. E2F1 binds to the promoters of the *miR-17~92* and *miR-106a~363* clusters and induces their transcriptional expression. Our findings present an E2F1-miR-20a-5p/20b-5p auto-regulatory feedback loop and suggest that this loop is important for myoblast proliferation and differentiation. Additionally, this auto-regulatory loop might also be involved in the regulation of *E2F1* expression during myoblast differentiation. These findings offer new insight into the mechanisms and regulatory networks of muscle differentiation, and further shed light on the understanding of muscle development and muscle diseases.

## Methods

### Cell culture

Chicken primary myoblasts were isolated and cultured as previously described^15^. QM-7 cells were cultured in Medium 199 (Gibco) with 10% (v/v) fetal bovine serum (Gibco), 10% tryptose phosphate broth (Sigma Life Science) and 0.2% penicillin/streptomycin (Invitrogen). When switched to medium without serum, the QM-7 cells cease dividing and fuse to form large multinucleated myotubes. DF-1 cells were cultured in high-glucose Dulbecco’s modified Eagle’s medium (Gibco) with 10% (v/v) fetal bovine serum (Gibco) and 0.2% penicillin/streptomycin (Invitrogen). All experimental protocols were approved by the South China Agricultural University Institutional Animal Care and Use Committee (approval ID: SCAU#0014). And the methods were carried out in accordance with the regulations and guidelines established by this committee.

### RNA isolation, RT-PCR and quantitative real-time PCR

RNAiso reagent (Takara) was used for isolating the total RNA from cells. cDNA synthesis was performed using PrimeScript^TM^ RT reagent Kit (Perfect Real Time) (Takara). qPCR was carried out using KAPA SYBR® FAST qPCR Kit (KAPA Biosystems) by Bio-rad CFX96 Real-Time Detection system (Bio-Rad). For miRNA quantitation, miRNA reverse transcription was performed using Bulge-loop miRNA qRT-PCR Primer Sets (RiboBio) and First-Strand cDNA Synthesis Kit (Fermentas) according to the manufacturer’s instructions. The qPCR primers specific for gga-miR-20a-5p, gga-miR-20b-5p and U6 were designed by RiboBio, and the qPCR was also performed using KAPA SYBR FAST qPCR Kit (KAPA). The quantification of qPCR results was done as previously described^15^.

### Western blot and immunofluorescence

Western blot and immunofluorescence assays were performed as previously described^15^. The following antibodies were used for Western blot: E2F1 (Santa Cruz Biotechnology) and Tubulin (Bioworld). The following antibody was used for immunoblotting: MyHC (DSHB). The total myotube area was calculated as the percentage of the total image area covered by myotubes, and the measurement was performed using ImageJ software (National Institutes of Health) on cells labelled with anti-MyHC.

### ChIP assays

ChIP assay was performed using Pierce Agarose ChIP Kit (Thermo Fisher Scientific) following the manufacturer’s instructions. Briefly, 10^6^ cells of the chicken primary myoblasts were fixed in 1% formaldehyde to cross-link DNA and proteins. After incubation of 10 min at 37 °C, 125 nM glycine was added to neutralization. Then, the cells were lysed and chromatin was sheared to 200–1000 bp DNA fragments using micronuclease digestion. Input DNA was stored for following assay. Remaining sheared DNA was incubated with anti-E2F1 antibody (Santa Cruz Biotechnology). Normal Rabbit IgG was used as negative control. The samples were then reversed and purified using a DNA clean-up column. ChIP products were finally subjected to normal PCR and quantitative PCR. Samples were done in triplicate. The primer sequences for PCR analysis are listed in Supplementary File 2.

### RNA oligonucleotides and transfection

The miR-20a-5p mimic, miR-20b-5p mimic, mimic control duplexes, miR-20a-5p inhibitor, miR-20b-5p inhibitor and inhibitor NC were purchased from RiboBio. siRNA against *E2F1* was purchased from GenePharma. Lipofectamine 3000 reagent (Invitrogen) was used for transfection according to the manufacturer’s instructions. 50 nM miRNA mimics, 100 nM miRNA inhibitor (as well as the mixed inhibitors) or 100 nM siRNA was used in the transfection assay.

### Plasmid construction

For *E2F1* overexpression vector, the *E2F1* coding sequences were amplified and inserted into the pcDNA-3.1 vector (Invitrogen) using the *Xho*I and *EcoR*I restriction sites. For pmirGLO dual-luciferase miRNA target reporter vector, the *E2F1* 3′ UTRs were amplified and inserted into the pmirGLO dual-luciferase reporter vector (Promega) using the *Dra*I and *Sal*I restriction sites. E2F1-3′ UTR mutant plasmid was generated by changing the binding site of miR-20a-5p and miR-20b-5p from CACTTT to AGAGCG. PCR amplification was performed to do the mutagenesis and *Dpn*I digestion to remove the parental DNA. For *miR-17~92* and *miR-106a~363* promoter reporter plasmids, four fragments of the *miR-17~92* and *miR-106a~363* promoters were amplified using the primers listed in Supplementary File 2. Then the PCR products of Reporter-1 and Reporter-2 were digested with *KpnI* and *SmaI*, the PCR products of Reporter-3 was digested with *SacI* and *SmaI*, and the PCR products of Reporter-4 was digested with *KpnI* and *XhoI*. After digestion, the products were inserted into the pGL3-basic reporter vector (Promega) to create the reporters of pGL3-R1, pGL3-R2, pGL3-R3 and pGL3-R4.

### Dual luciferase reporter assay

DF-1 cell, an immortalized cell line of chicken embryo fibroblasts^52^, was chosen to do the reporter experiment because of its more stable status than the QM-7 cell. The dual luciferase reporter assay was performed as previously described^15^. Briefly, for the promoter assays of pri-miRNAs, DF-1 cells were co-transfected with reporter plasmid and E2F1 overexpression vector or control vector, and the TK-Renilla reporter was also co-transfected to each sample as an internal control. For miRNA target validation assays, DF-1 cells were co-transfected with wild-type or mutant E2F1 3′UTR dual-luciferase reporter (100 ng) with miRNAs mimic or NC mimic (50 nM) by using Lipofectamine 3000 reagent (Invitrogen) in 96-well plates. The activities of luciferase were measured using Dual-luciferase reporter assay system according to the manufacturer’s instructions (Promega) after 36 h transfection. The luminescent signal was quantified using Synergy 2 Multi-mode Microplate Reader (Biotek) and analysed with Gene5 software (Biotek).

### Cell cycle analysis

After 36 h transfection, QM-7 cells cultured in growth medium were collected, fixed in 75% ethanol overnight at −20 °C, and then stained with 50 μg/mL propidium iodide (Sigma) containing 10 μg/mL RNase A (TaKaRa) and 0.2% (v/v) Triton X-100 (Sigma) for 30 min at 4 °C. Subsequently the cell cycle were analysed using a BD Accuri C6 flow cytometer (BD Biosciences), and the data analysis was performed using FlowJo 7.6 software (Verity Software House).

### CCK-8 assays

The transfected myoblasts were seeded in 96-well plates (1 × 10^3^ cells/well) and cultured in growth medium. Every 12 h, 10 μL of Cell-Counting Kit-8 (CCK-8) solution (Dojindo Laboratories) was added to each well for 1 h, and absorbance was measured at 450 nm using a Model 680 Microplate Reader (Bio-Rad).

### Statistical analysis

Each experiment was repeated three times, and all results are represented as mean ± sem. One-sample *t* test was used to perform the statistical significance test between groups.

## Additional Information

**How to cite this article**: Luo, W. *et al*. E2F1-miR-20a-5p/20b-5p auto-regulatory feedback loop involved in myoblast proliferation and differentiation. *Sci. Rep.* **6**, 27904; doi: 10.1038/srep27904 (2016).

## Supplementary Material

Supplementary Information
