# Supplementary material for: E2F1-miR-20a-5p/20b-5p auto-regulatory feedback loop involved in myoblast proliferation and differentiation
Source: Sci Rep. 2016 Jun 10;6:27904. doi: 10.1038/srep27904 (PMC4901305; doi:10.1038/srep27904)
Supplement: Supplementary Information [file srep27904-s1.pdf]

E2F1-miR-20a-5p/20b-5p auto-regulatory feedback loop involved in myoblast proliferation and differentiation

Wen Luo[1, 2], Guihuan Li[1, 2], Zhenhua Yi[1, 2], Qinghua Nie[1, 2], Xiquan Zhang[*1, 2]

Supplementary file 1. The histograms of cell cycle analyzed by flow cytometry.

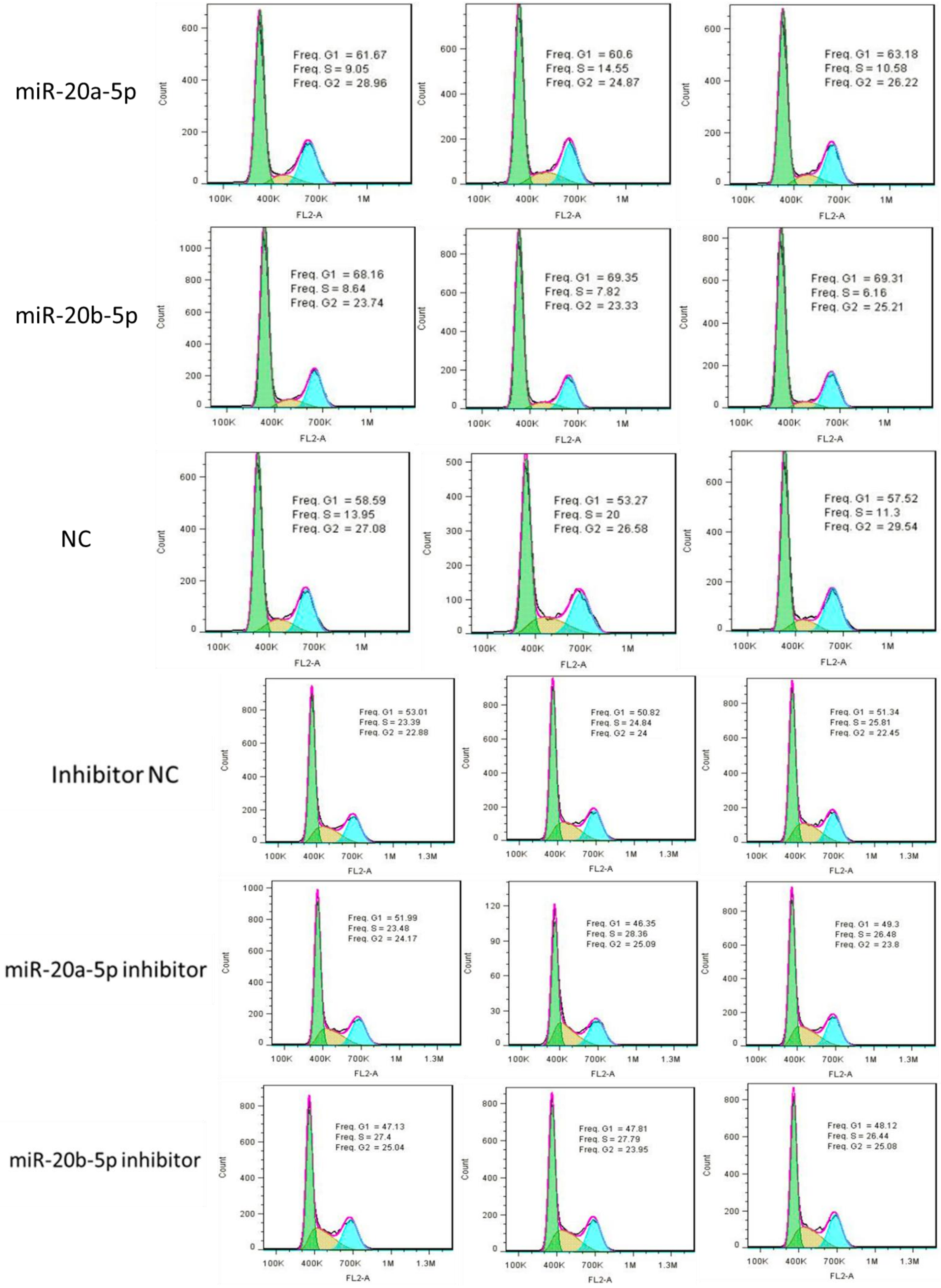

miR-20a-5p/20b-5p and pcDNA3.1-EGFP

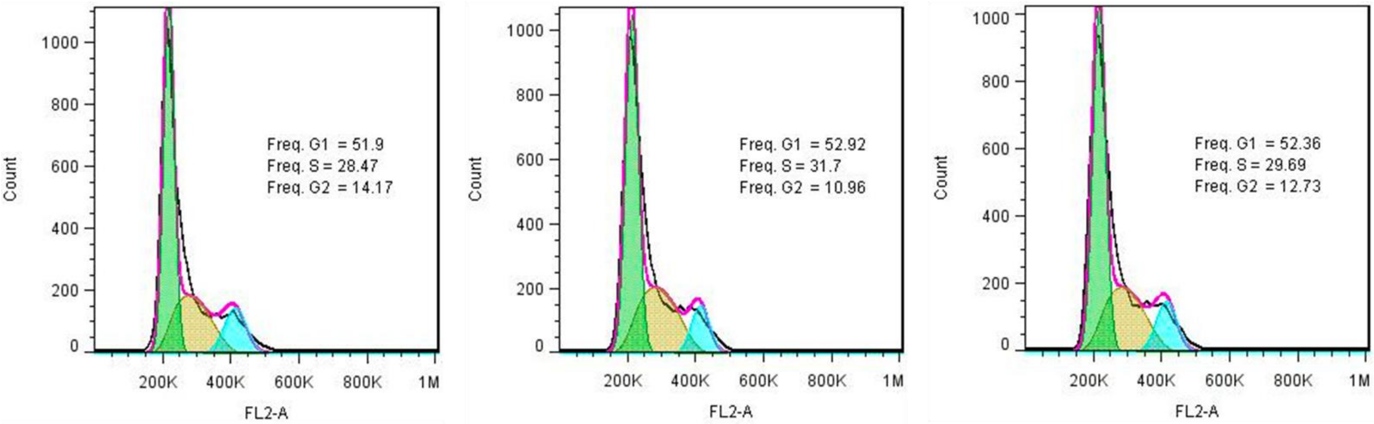

miR-20a-5p/20b-5p and pcDNA3.1-E2F1

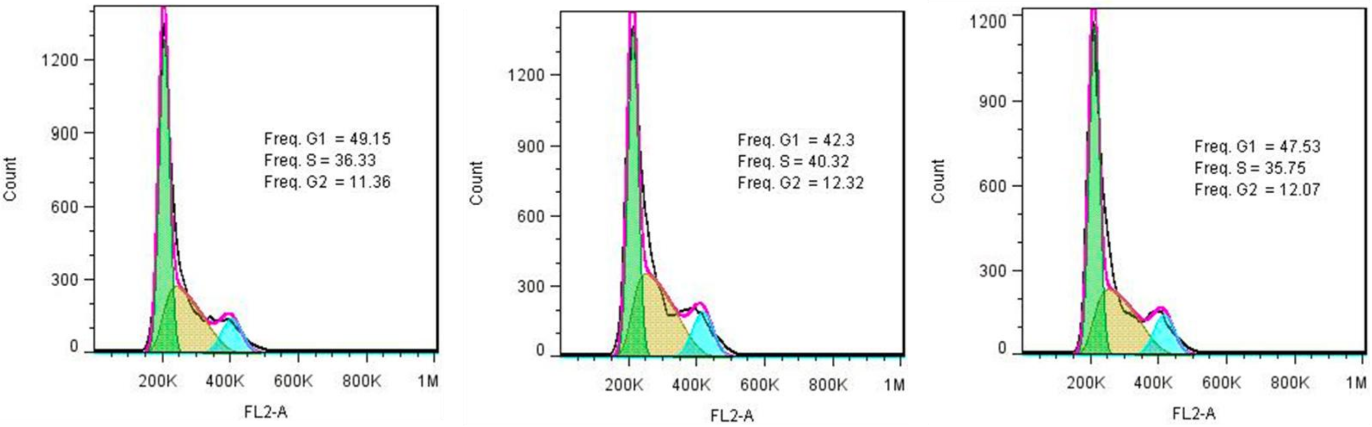

NC and pcDNA3.1-EGFP

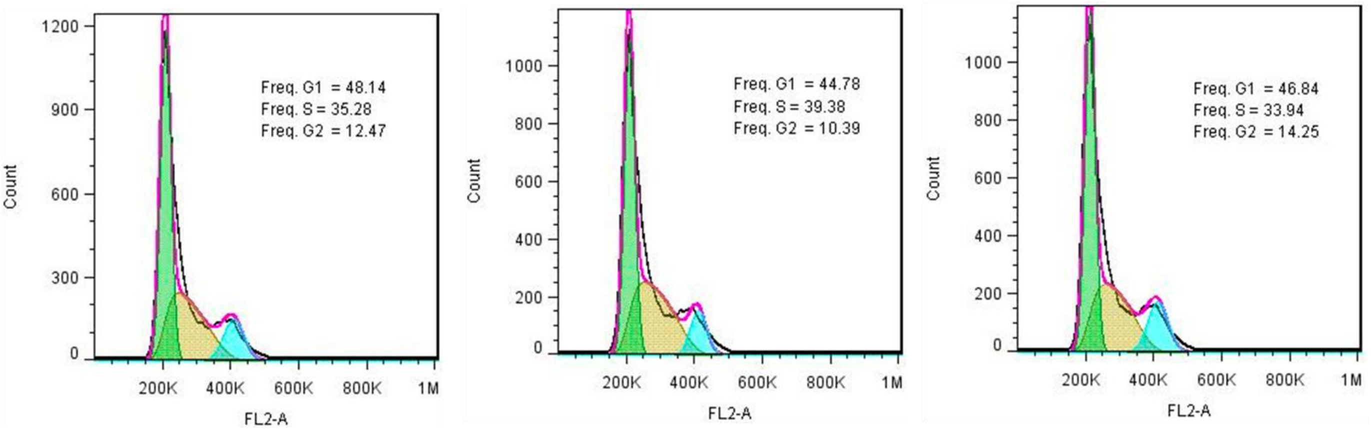

pcDNA3.1-E2F1

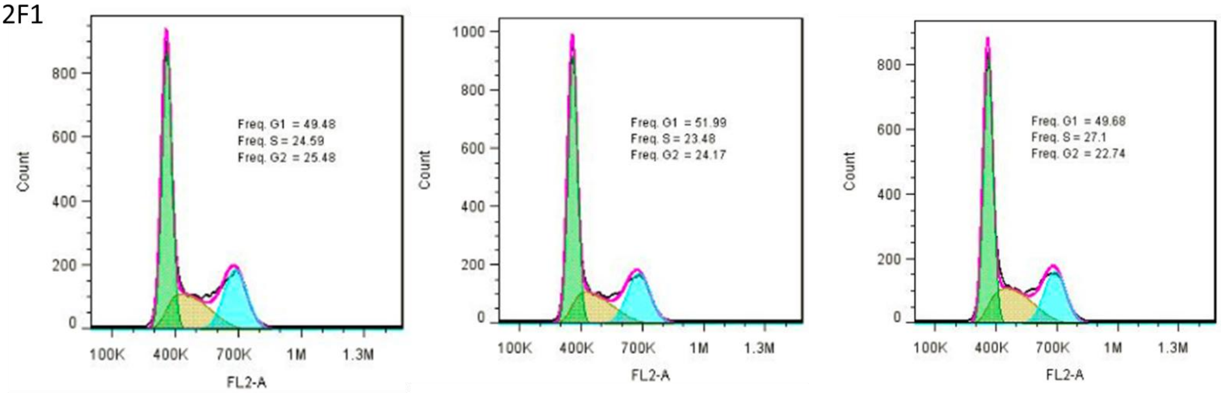

pcDNA3.1-EGFP

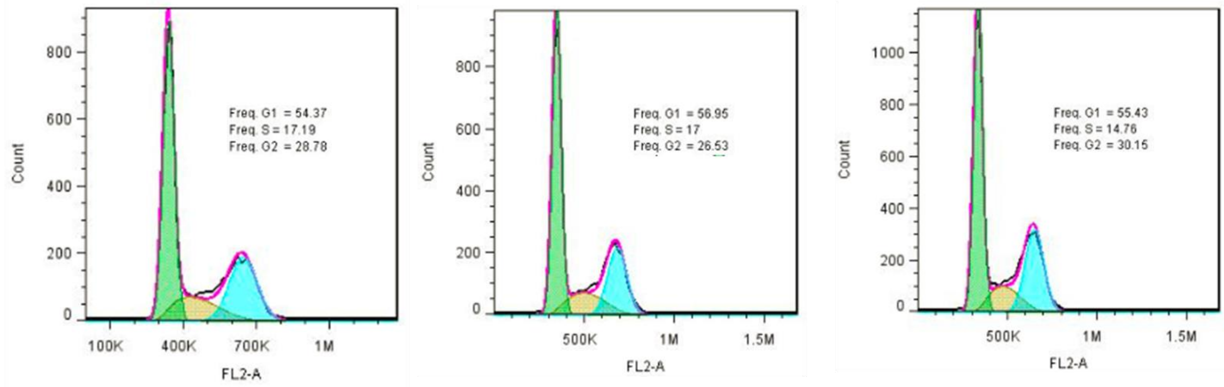

si-E2F1

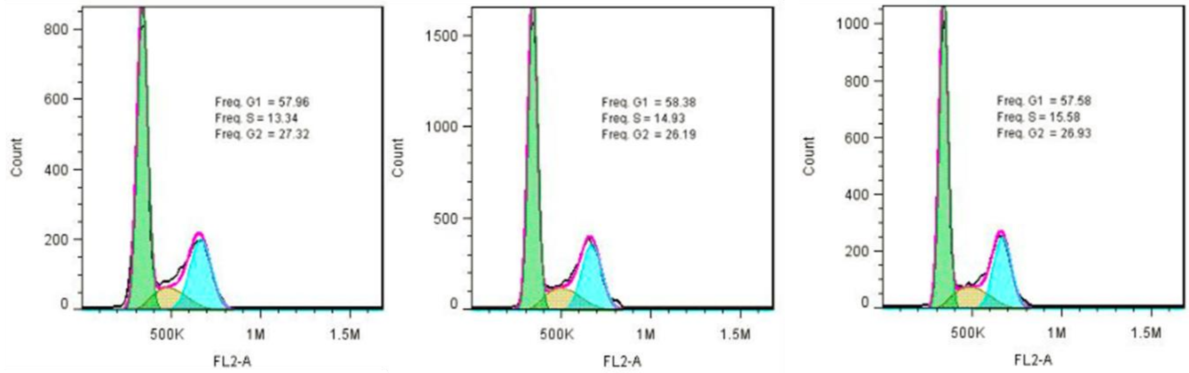

si-NC

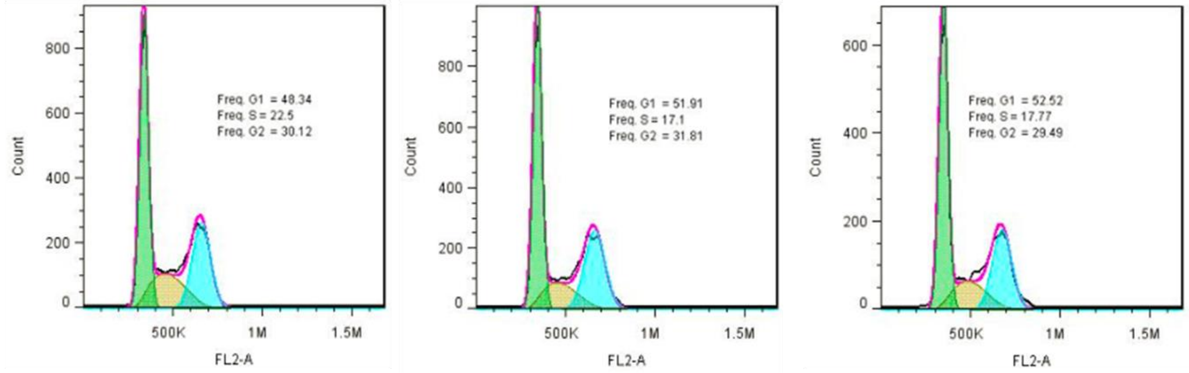

Supplementary File 2.

Oligonucleotides:
For ChIP-qPCR or PCR analysis the following primers were used:
Amplico A:
s: 5’- GCACTTGTCCGAGCGGGAATC
as: 5’- CAGTCGCAGCTCATGGTGGC
Amplico B:
s: 5’ - TACTCGCTTTGGGTTGCTG
as: 5’ - CGGACGGCTGCTGGTTT
Amplico C:
s: 5’ - AAACAGTTGTATGGGTGA
as: 5’ - GACAGGGTCTTATTATGC
Amplico D:
s: 5’ - CCGGTCCTGCCATGTTT
as: 5’ - ACGCTTCCACTGCTCCTG
Amplico E:
s: 5’ - GAGAATGGAAAGATAAACACGG
as: 5’ - TTTTGGCTGGTCGGTGC

For cloning the following primers were used for PCR:
E2F1 CDS (reference sequence: NM_205219.1) cloning for pcDNA3.1:
s: 5’- GGGGTACCCCCGTCACCTGAGCCATG
as: 5’- CCGCTCGAGGAGGAGGAAACAAACCCAAAA
Reporter-1 cloning: s: 5’- cggGGTACCGGGAAAGGGAGGTCTCG
as: 5’- tccCCCGGGGGCTGTAAGTTACCCAAGC
Reporter-2 cloning: s: 5’- cggGGTACCAACCACCACAACACGCAATG
as: 5’- ccgCTCGAGATGTTGACCTCTCGCCATCG
Reporter-3 cloning: s: 5’- cGAGCTCTTTGGCTGGTCGGTGCG
as: 5’- tccCCCGGGGCGAAAGAAAAGAAAGAAAGG
Reporter-4 cloning: s: 5’- cggGGTACCAGAAGGGAAGAGTGAGCCG
as: 5’- ccgCTCGAGTCGCAGGAATGCCAAAA
E2F1 3’UTR Fragment (reference sequence: NM_205219.1):
s: 5’- CCGCTCGAGTGCCTCACTGCCAAGGG
as: 5’- ACGCGTCGACCGAAGCTCAGCGGAACAC

For mutagenesis the following primers were used for PCR:
E2F1-3’UTR-mut:
s: 5’- AGGCCCCGGGGCCAGGCTGGAGCCGAGAGCGCACTTACTGCCTTTAACAAGCTTGT
as: 5’- ACAAGCTTGTTAAAGGCAGTAAGTGCGCTCTCGGCTCCAGCCTGGCCCCGGGGCCT

For quantitative real time RT-PCR, the following primers were used:
Myomaker-qPCR (reference sequence: KP230536.1): s: 5’- TGGGTGTCCCTGATGGC
as: 5’- CCCGATGGGTCCTGAGTAG
MYOD-qPCR (reference sequence: NM_204214.2): s: 5’- GCTACTACACGGAATCACCAAAT
as: 5’- CTGGGCTCCACTGTCACTCA
MYOG-qPCR (reference sequence: NM_204184.1): s: 5’- CGGAGGCTGAAGAAGGTGAA
as: 5’- CGGTCCTCTGCCTGGTCAT
MyHC-qPCR (reference sequence: ENSGALT00000001427):

s: 5’- CTCCTCACGCTTTGGTAA
as: 5’- TGATAGTCGTATGGGTTGGT
E2F1-qPCR (reference sequence: NM_205219.1): s: 5’- AGACGGATCACCAGTACATAGCC
as: 5’- GTTCAGCGAGGTTTCATAGCG
β-actin-qPCR (reference sequence: NM_205518.1): s: 5’- TTGTTGACAATGGCTCCGGT
as: 5’- AACCATCACACCCTGATGTCT
pri-miR-17~92-qPCR (reference sequence: chicken genome, chr1: 147252628-147254423):
s: 5’- CCTGAGGCAGCTGTCAACAT
as: 5’- AAAGCTGGTTGCGTCAGAGT
pri-miR-106a~363-qPCR (reference sequence: chicken genome, chr4: 3945818-3947210):
s: 5’- TGTGGAGGAAAGGAGGAC
as: 5’- TTGCGGTTTACAGATGGA

Supplementary File 3.

The used in the main figures of full-gels are shown. All cropped gels have been run under the same experimental condition.

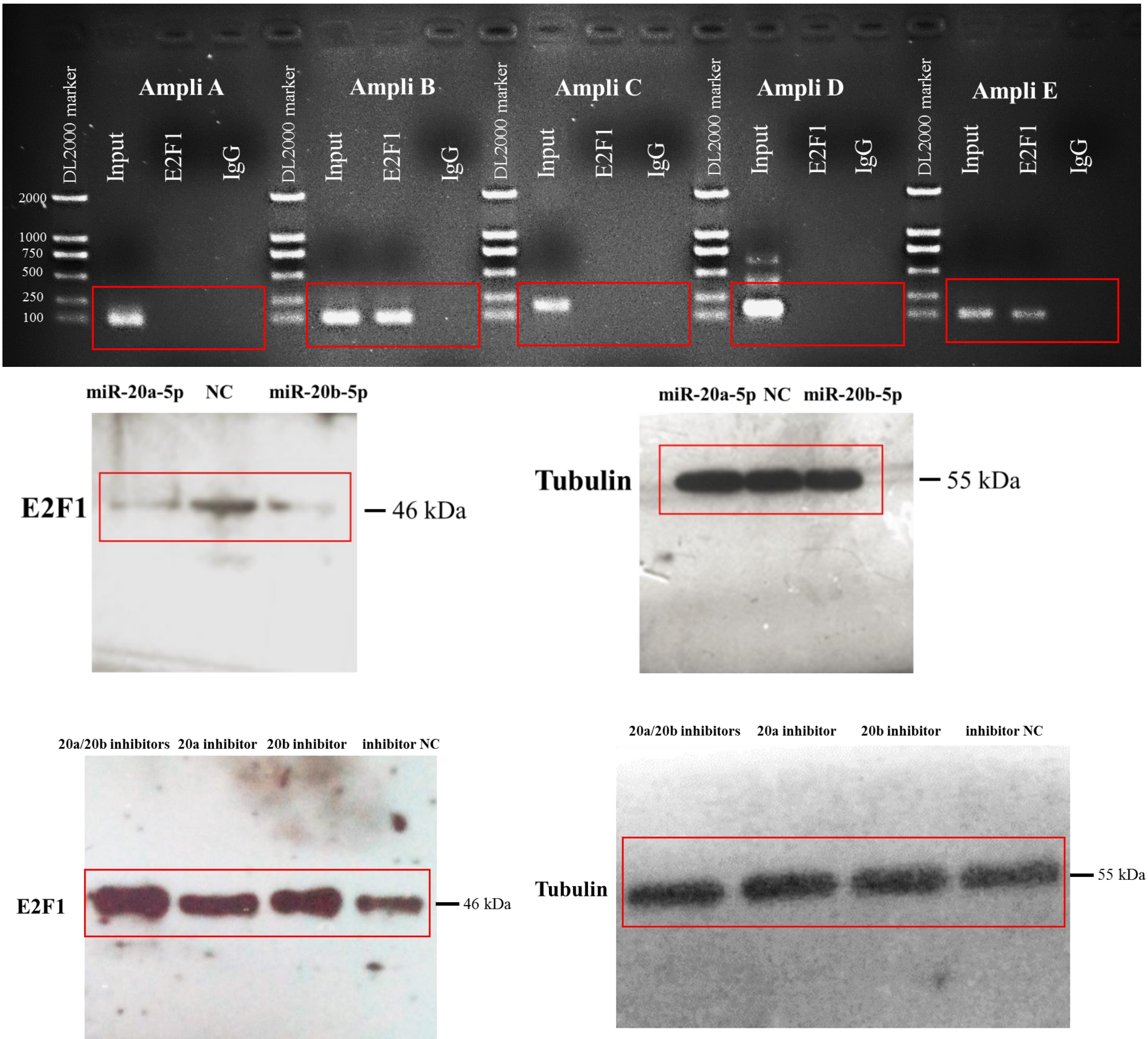

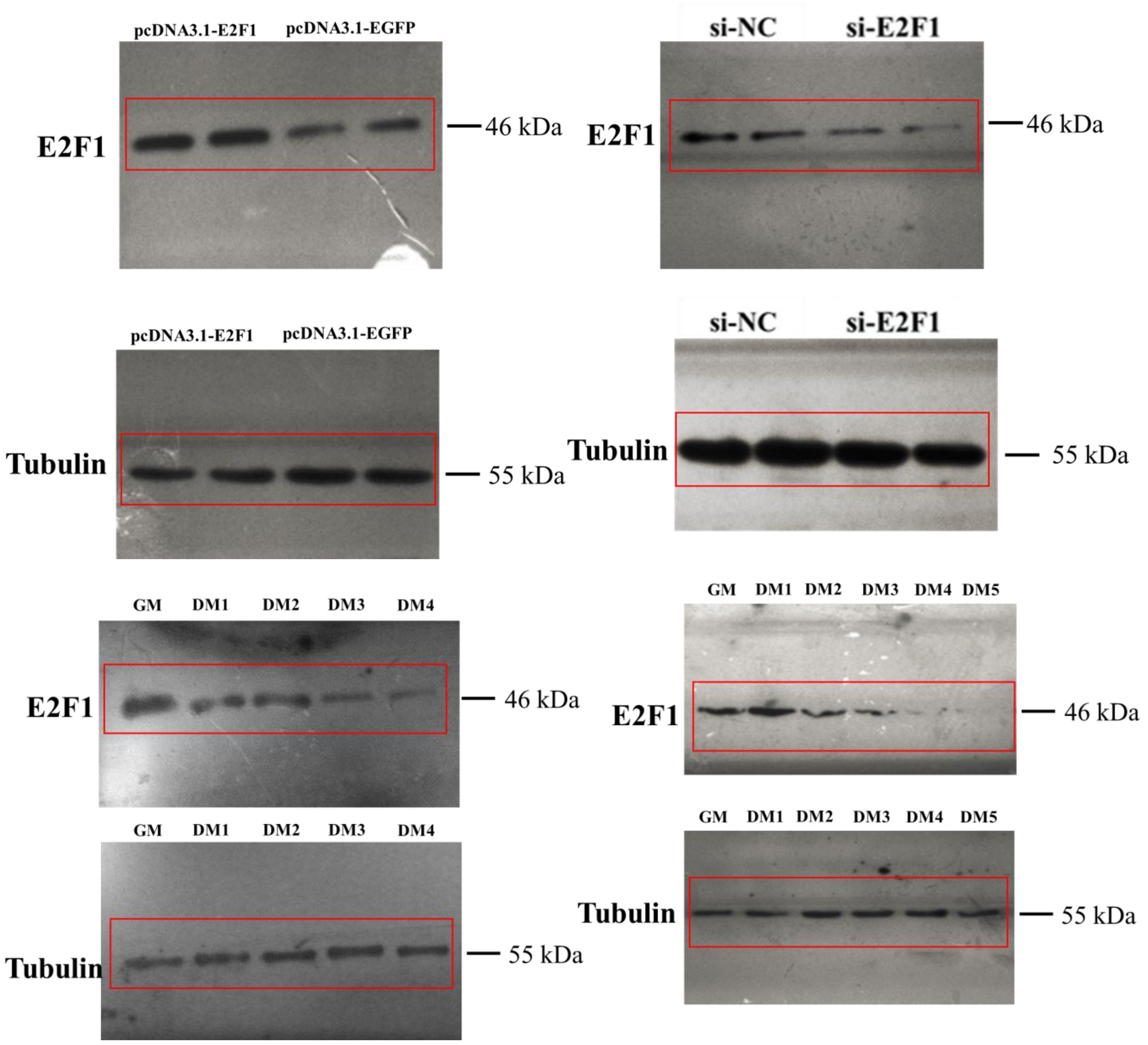
pcDNA3.1-E2F1
pcDNA3.1-EGFP
E2F1
46 kDa
si-NC
si-E2F1
E2F1
46 kDa
pcDNA3.1-E2F1
pcDNA3.1-EGFP
Tubulin
55 kDa
si-NC
si-E2F1
Tubulin
55 kDa
GM
DM1
DM2
DM3
DM4
E2F1
46 kDa
GM
DM1
DM2
DM3
DM4
DM5
E2F1
46 kDa
GM
DM1
DM2
DM3
DM4
Tubulin
55 kDa
GM
DM1
DM2
DM3
DM4
DM5
Tubulin
55 kDa
